# A New Strategy for the High-Value Utilization of Cobalt Slag: A Solid-State Reaction for the Preparation of Microwave-Absorbing Composite Materials with Excellent Properties

**DOI:** 10.3390/ma18061373

**Published:** 2025-03-20

**Authors:** Xuanzhao Shu, Zeying Wang, Rifan Chen, Yangyang Fan

**Affiliations:** 1 Zhongyuan Critical Metals Laboratory, Zhengzhou University, Zhengzhou 450001, China; xz_shu@163.com (X.S.); 15709459691@163.com (Z.W.); 2 Key Laboratory of Critical Metals Minerals Supernormal Enrichment and Extraction of Ministry of Education, Zhengzhou University, Zhengzhou 450001, China; 3 Laibin Smelter, Guangxi Huaxi Group Co., Ltd., Laibin 546100, China; chenrifan2023@126.com; 4 School of Materials Science and Engineering, Zhengzhou University, Zhengzhou 450001, China

**Keywords:** cobalt slag, waste utilization, solid-phase reaction, MnCo_2_O_4_, graphene composites, microwave absorption

## Abstract

Abundant valuable metals such as manganese and cobalt are present in cobalt-rich slags from the hydrometallurgical zinc industry. However, due to the high cost of traditional hydrometallurgical separation methods, these metals cannot be effectively recovered. In this paper, a novel recycling strategy based on mineral phase recovery was proposed, utilizing cobalt-rich slags as raw materials to fabricate microwave-absorbing composite materials. A feasible solid-phase thermochemical method has been developed to recover the mineral phase from cobalt-rich slags, with calcination temperature 800 °C and duration 90 min, yielding MnCo_2_O_4_ spinel. The results demonstrated that under the conditions of a ball-to-material ratio of 20:1 and ball milling time of 4 h, the MnCo_2_O_4_ powder and graphene materials, after being ball-milled and compounded, exhibited appropriate electromagnetic parameters and impedance matching. At 5.2 GHz, the minimum reflection loss of the composite material reached −40 dB. This study provides a new approach for the value-added utilization of valuable metal resources in cobalt-rich slags.

## 1. Introduction

The cobalt slag originating from the hydrometallurgical zinc industry is a type of heavy-metal-containing solid waste. It is formed as a metallurgical slag with a high cobalt content during the purification of zinc sulfate electrolytes in the hydrometallurgical zinc process in order to enhance current efficiency and the quality of electrolytic zinc [1]. In addition to the abundant elements of Co and Zn, the cobalt slag also contains certain amounts of valuable metals such as Mn, Ni, and Cd, as well as non-metallic elements like As and S.

At present, the recovery of cobalt slag from the hydrometallurgical zinc industry primarily adheres to the principles of traditional mineral processing and metallurgy, with the objective of removing zinc from the cobalt slag and separating the cobalt from other valuable metals. The cobalt recovery process typically involves a combination of various operations, including the following: (1) selective leaching [2], which uses the acidity of the solution to selectively dissolve most of the zinc in the cobalt slag, while the leaching rate of Co does not exceed 6.24%; (2) the ammonium sulfate method [3], which achieves the dissolution of Co, Zn, and Cu in the cobalt slag in the solution system, with a cobalt leaching rate exceeding 95%, while Fe and Sb are removed in the form of hydroxide precipitates; (3) oxidation precipitation [4], which uses a strong oxidizing agent to oxidize Co^2+^ in the leachate to Co^3+^, causing Co to hydrolyze and form precipitates, with a cobalt recovery rate of over 88%; and (4) solvent extraction [5], which uses a combination of various extractants to achieve the sequential separation of Zn, Fe, Cu, Co, and Mn in the leachate. Huang [6] selected an alkaline glycine solution as the leaching agent, selectively dissolving metals such as Zn and Cd from the cobalt slag, achieving dissolution rates of 93.9% for Zn, 87.64% for Cd, 2.05% for Co, and 0.42% for Mn. Liu [7] discovered that after acid leaching and oxidative iron removal from cobalt slag, the addition of ammonium persulfate in excess of the cobalt content, followed by adjusting the pH to 4.5–5.2 with a sodium carbonate solution and reacting for 1–2 h and subsequent acid washing, could yield a high-grade cobalt slag with less than 10% Zn content and over 30% Co content. Due to the diverse elemental composition of cobalt-rich slag, the further purification of cobalt inevitably faces the issue of impurity removal. The cobalt-rich slag needs to undergo further acid leaching and multi-stage extraction and separation of elements such as Zn, Co, Cu, and Mn in the leachate through extractants like P204, D2EHPA, and LIX 984, inevitably leading to long process durations and high reagent costs [3,5]. Therefore, the efficient utilization of cobalt-rich slag holds significant research value in addressing the storage and treatment issues of such harmful substances.

The solid-phase reaction method, also known as the ceramic method, is a preparation technique where raw materials are mixed and then calcined to undergo solid-phase reactions [8,9]. This method can directly yield ultrafine powders or produce them after grinding the calcined products. Compared to wet synthesis processes, solid-phase reactions do not require the use of liquid solvents, offering advantages such as a high selectivity, high yield, and simplicity of process. This method has been widely applied in the research and preparation of materials such as the magnetic material MnFe_2_O_4_ [10] and the electrode material NiCo_2_O_4_ [11]. Cobalt-rich slag is rich in Mn and Co. Utilizing cobalt-rich slag as a raw material and forming uniform composite oxides through solid-phase reactions represents a promising approach for the high-value utilization of cobalt-rich slag.

Currently, the rapid development of electronic products has led to increasingly severe electromagnetic pollution [12,13]. Graphene-based cobalt oxide materials, due to their excellent reflection loss, have been extensively studied for mitigating electromagnetic pollution [14,15,16]. However, the development of these microwave-absorbing materials still faces significant challenges, such as complex material production processes and limited product quantities. The preparation of cobalt metal oxide microwave-absorbing materials typically follows wet chemical synthesis technical routes, including precipitation [17], sol–gel [18], hydrothermal/solvothermal [19], and electrospinning methods [20]. These methods generally use pure metal salts as raw materials and, after the dissolution of single or multiple elements in a solvent system, a precursor with specific morphological structures and chemical compositions is obtained by adding co-precipitants, controlling reaction conditions, or applying electrochemical environments, and then the metal oxide is obtained after calcination [21,22]. Masoudpanah [23] prepared spinel cobalt manganate using oxalic acid as a precipitant. When the matching thickness was 2.4 mm, the absorption rate of MnCo_2_O_4_ powder for the X-band reached 60%, with a minimum reflection loss of −43 dB. Chen [24] prepared Co_3_O_4_/rGO composite materials from graphite powder and cobalt acetate using a simplified hydrothermal method. The reflection loss and effective absorption bandwidth of the Co_3_O_4_/rGO composites could be adjusted by changing the thickness and compositional ratio of the material. Ding [25] successfully loaded Co nanoparticles onto graphene via hydrothermal synthesis, and then prepared Co_x_O_y_/rGO composite materials through pyrolysis. The minimum reflection loss of the composite material reached −67.2 dB, with an effective absorption bandwidth of 9.1 GHz, achieving coverage of the entire X, Ku, and K frequency bands. Although these methods can yield microwave-absorbing materials with good microwave absorption performance, the use of high-purity metal salts as raw materials results in a high technical difficulty of production and cost, which greatly limits the application of cobalt oxide microwave-absorbing materials. Therefore, changing the production process, expanding the sources of raw materials, and improving production efficiency are of great significance for the industrial production and microwave-absorbing applications of cobalt metal oxide materials.

Considering the elemental composition and functional application characteristics of spinel MnCo_2_O_4_ materials, as well as the metal element composition in cobalt-rich slag from hydrometallurgical zinc refining, this study used cobalt-rich slag from a hydrometallurgical zinc enterprise as the raw material to investigate the process of solid-phase reactions for preparing MnCo_2_O_4_ materials and their microwave-absorbing applications. The aim was to provide a new approach for directly preparing MnCo_2_O_4_ materials from cobalt-rich slag, thereby offering new insights into the value-added utilization of valuable metals in secondary resources such as cobalt-rich slag. The main tasks include the following: (1) achieving the transformation of cobalt slag into MnCo_2_O_4_ materials by adjusting the calcination conditions; (2) enhancing and optimizing the microwave absorption properties of MnCo_2_O_4_ through ball milling and composite techniques.

## 2. Materials and Methods

### 2.1. Materials

During the experimental process, graphene was purchased from TCI (Shanghai, China) Development Co., Ltd. The cobalt-rich slag was sourced from a hydrometallurgical zinc enterprise in Henan Province, China. As shown in Table 1, the cobalt-rich slag primarily contained metallic elements such as Co, Mn, Zn, Cd, and Cu, with high grades of cobalt and manganese, at 22.01% and 19.64%, respectively. Owing to the presence of a certain amount of soluble zinc in the cobalt-rich slag, a water-washing treatment was conducted to ensure the stability of the raw material composition during the solid-phase reaction process.

### 2.2. Synthesis of MnCo_2_O_4_ Materials

The experimental procedure for the solid-phase reaction of the cobalt-rich slag was primarily divided into three steps: pretreatment, powder compaction, and tube furnace calcination. Initially, the cobalt-rich slag was mechanically stirred and washed in boiling water at 90 °C for 120 min, a process repeated three times to remove soluble impurities from the slag. The washed slag was then dried at 80 °C for 60 min for compaction. Subsequently, an automatic tablet press was employed to compact the powdered slag into cylindrical tablets with a diameter of 20 mm and a height of 6 mm. The samples were placed in a tubular furnace for solid-phase calcination under a programmed temperature regime. The heating rate of the tube furnace was maintained at 5 °C/min. The calcination experiment was conducted in an air atmosphere, with the samples cooling to room temperature with the furnace. The cooled samples were ground to a particle-free consistency for characterization and subsequent experiments.

### 2.3. Synthesis of MC@G Composites

The MnCo_2_O_4_ material (800 °C, 90 min) was derived from the solid-phase calcination experiment of the cobalt-rich slag. The construction of the composite phase between the cobalt manganate and graphene was accomplished using a high-energy planetary ball mill. The mass ratio of MnCo_2_O_4_ to graphene was 1:1. The ball milling media consisted of zirconia balls (φ = 7 mm, φ = 4 mm, φ = 2 mm), with a ball-to-material ratio of 20:1 and a ball milling duration of 4 h. The resulting sample was denoted as MC@G.

### 2.4. Characterizations

In this study, the phase analysis of samples was conducted using a Rigaku SmartLab SE X-ray diffractometer (XRD; Tokyo, Japan). The instrument utilized Cu Kα radiation as the X-ray source, with a tube voltage and current of 40 kV and 30 mA, respectively. The step size was set at 0.01° (2θ), with a scanning range of 10–80° and a scanning speed of 4°/min. The surface morphology and microstructure of the samples were analyzed using a ZEISS Sigma300 scanning electron microscope (SEM; Oberkochen, Germany). The operating voltage and current were 12.5 kV and 16 mA, respectively. The magnetic hysteresis loops of the experimental samples were tested using a Lake Shore 7404 vibrating sample magnetometer (VSM; Westerville, OH, USA). The chemical bonds present in the samples were analyzed using a Thermo Scientific Nicolet iS20 Fourier transform infrared spectrometer (FT-IR; Waltham, MA, USA)with a testing wavelength range of 400–4000 cm^−1^. The chemical environment of the metal atoms in the samples and the degree of graphitization of the carbon in the composite materials were investigated using a HORIBA Scientific LabRAM HR Evolution Raman spectrometer (Kyoto, Japan) with a laser wavelength of 532 nm. X-ray photoelectron spectroscopy (XPS) was conducted using an instrument from ThermoFischer Inc., located in Waltham, MA, USA. The analysis was performed in a vacuum of 8 × 10^−10^ Pa, using Al Kα radiation (hv = 1486.6 eV) as the excitation source. The operating voltage was 12.5 kV and the filament current was 16 mA. The pass energy was set at 100 eV for full-spectrum analysis and 20 eV for narrow-spectrum analysis, with a step size of 0.05 eV and a dwell time of 40–50 ms. The specific surface area and pore size distribution of the metal oxide raw materials were determined using a MicrotracBEL BELSORP MAX adsorption analyzer (Ōsaka shi, Iapan) at 77 K. The calculations were based on the Brunauer–Emmett–Teller (BET) multilayer adsorption theory and the Barret–Joyner–Halenda (BJH) theory. The electromagnetic parameters of the samples in the frequency range of 2–18 GHz were measured using an Keysight E5071C vector network analyzer (Santa Rosa, CA, USA) with the coaxial line method. For the preparation of samples for electromagnetic parameter testing, the experimental samples were mixed with melted paraffin at a mass ratio of 1:1. After cooling the mixture to room temperature, it was placed into a mold and pressed into test samples (inner diameter φ_in_ = 3 mm, outer diameter φ_out_ = 7 mm) using a tablet press.

## 3. Results and Discussion

### 3.1. Characterization of Cobalt-Rich Slag

**Figure 1:**
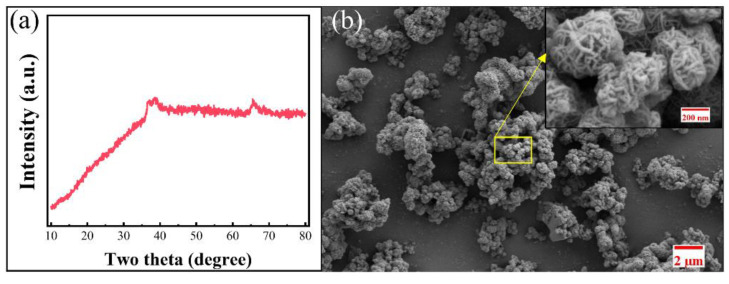
XRD pattern (**a**) and SEM images (**b**) of cobalt slag with abundant cobalt in zinc hydrometallurgy.

The XRD pattern of the cobalt-rich slag raw material is shown in Figure 1a. The X-ray diffraction results indicate that no distinct substance phases correspond to the characteristic diffraction peaks of the cobalt-rich slag raw material, suggesting that the metal elements in the slag have not yet formed compounds with clear crystalline structures. The apparent morphology of the cobalt-rich slag raw material was examined using a SEM, and the results are presented in Figure 1b. The cobalt-rich slag particles are composed of microspheres with a diameter of approximately 200 nm, and the surface structure of the individual microspheres is complex. The complex morphology facilitates contact between metal atoms, thereby promoting solid-phase reactions.

**Figure 2:**
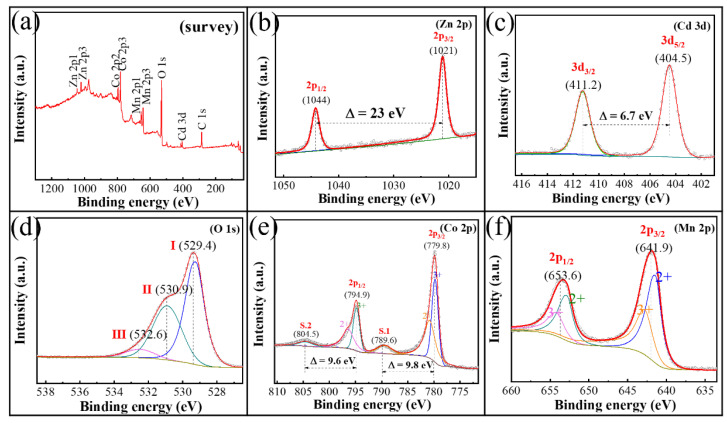
XPS spectra of cobalt slag: (**a**) Survey, (**b**) Zn 2p, (**c**) Cd 3d, (**d**) O 1s, (**e**) Co 2p, (**f**) Mn 2p.

The existence state of the metal ions determines the phase transformation process and the formation mechanism of calcined products during the solid-phase calcination process. The XPS analysis results of the cobalt-rich slag are shown in Figure 2. As can be seen from Figure 2a, in addition to the strong absorption peaks of C and O elements, the absorption peaks of the metal elements Mn, Co, Zn, and Cd are also significant, which is consistent with the elemental content analysis results of the slag. To determine the oxidation states of the metal elements in the slag, peak fitting was performed separately, and the results are shown in Figure 2b–f. The Zn element shows distinct 2p_3/2_ and 2p_1/2_ peaks at 1021 eV and 1044 eV, respectively, indicating that the Zn element exists in the +2 oxidation state [26]. Similarly, the peak fitting results of the Cd element show that the Cd element also exists in the +2 oxidation state [27]. The Co and Mn elements exhibit mixed valence states, coexisting in the +2 and +3 oxidation states in the slag [28,29]. Through the above analysis, it can be determined that the Zn and Cd atoms in the cobalt-rich slag from hydrometallurgical zinc refining exist in stable oxidation states, while the Co and Mn atoms possess multiple oxidation forms. Moreover, the metal elements are present on nanoscale microspheres that have not formed distinct phases.

### 3.2. Preparation of MnCo_2_O_4_ and MC@G

#### 3.2.1. Analysis of the Solid-Phase Reaction in Cobalt-Rich Slag

Based on the formation temperature of MnCo_2_O_4_ [28], solid-phase calcination experiments were conducted on cobalt-rich slag within the temperature range of 400 °C to 1000 °C (for 90 min).

**Figure 3:**
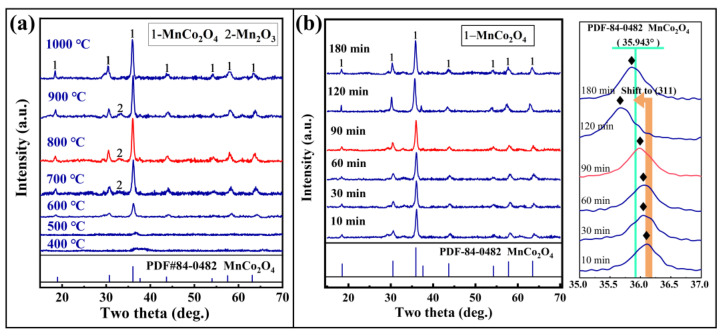
XRD results of cobalt slag roasted in air atmosphere: (**a**) temperature (90 min), (**b**) time (800 °C).

The results of the phase transformation of the calcined products are shown in Figure 3a. When the solid-phase reaction temperature is below 600 °C, the XRD results of the calcined products show weak diffraction peak intensities, indicating that the Mn and Co elements in the products still exist as a mixture of single metals or oxides in the form of metallurgical waste slag, without forming MnCo_2_O_4_. As the temperature increases, at a calcination temperature of 600 °C, the diffraction peak intensities of the calcined products are enhanced, and the 2θ positions of the diffraction peaks are consistent with the spinel structure of MnCo_2_O_4_, indicating that with the progress of the solid-phase reaction, the cobalt-rich slag gradually transforms into MnCo_2_O_4_ at the high temperature of 600 °C. Further increasing the calcination temperature to 800 °C, the diffraction peak intensities of the calcined samples are further enhanced, and the product phases become clearer. Additionally, it can be observed that in the calcined samples below 1000 °C, Mn_2_O_3_ appears in the products. According to the thermal stability studies of manganese oxides, it can be determined that at this time, manganese oxides mainly exist stably in the form of the Mn_2_O_3_ phase. This also proves that when preparing MnCo_2_O_4_ through the solid-phase reaction of cobalt-rich slag, MnCo_2_O_4_ is formed with Mn_2_O_3_ as the effective reactant, providing manganese ions to replace cobalt ions at the tetrahedral and octahedral positions of cobalt oxides, thereby forming a mixed spinel structure of MnCo_2_O_4_ [28]. Moreover, the thermal stability of the MnCo_2_O_4_ prepared by the solid-phase reaction method is exceptionally excellent.

Simultaneously, the phase transformations of the calcined products of the cobalt-rich slag at different calcination times (at 800 °C) were investigated, as shown in Figure 3b. Under the calcination temperature of 800 °C, the calcined products formed a clear MnCo_2_O_4_ phase in a short time. Further extending the calcination time to 90 min did not result in significant changes in the product phase, but the diffraction peak of the (311) crystal plane showed a noticeable shift, which may be related to the improvement of the MnCo_2_O_4_ lattice structure. To effectively transform cobalt-rich slag into MnCo_2_O_4_, appropriately extending the calcination time is beneficial for the formation of the spinel structure. Additionally, the material can maintain its spinel structure without decomposition even after prolonged calcination at high temperatures, indicating good thermal stability for microwave absorption applications.

**Figure 4:**
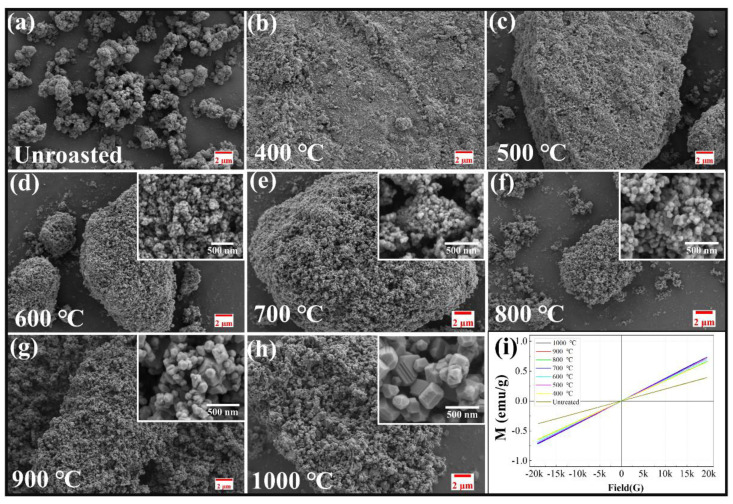
SEM images (**a–h**) and VSM (**i**) spectrum of calcined products of cobalt-rich slag at different temperatures.

To investigate the morphological evolution of the products and the characteristics of the MnCo_2_O_4_ during the calcination process, analysis was conducted on the calcined products at different temperatures using a SEM, as shown in Figure 4a–h. Compared to the dispersed granular cobalt-rich slag that has not undergone calcination (Figure 4a), the calcined products primarily exhibit micron-sized large particles without a fixed shape. In conjunction with the phase analysis of the calcined products, Figure 4d–h illustrates the morphological evolution of the MnCo_2_O_4_ during the solid-phase calcination process. It can be observed that the materials are composed of nanoscale MnCo_2_O_4_ particles, with severe agglomeration occurring between the particles. As the calcination temperature increases to 900 °C, the size of the small particles grows rapidly, and their shape evolves from spherical to regular polyhedral particles. The magnetic properties of the calcined products during the solid-phase reaction process are shown in Figure 4i. According to the classification of material magnetism, the VSM curve characteristics of the calcined samples indicate that calcination does not alter the magnetic properties of the products. The MnCo_2_O_4_ materials obtained through calcination are paramagnetic materials, and their coercivity and saturation magnetization are not considered.

#### 3.2.2. Physical Property Analysis of MnCo_2_O_4_

Based on the phase transformation and morphological evolution during the solid-phase reaction of cobalt-rich slag, to ensure the integrity of the spinel structure of the MnCo_2_O_4_ and maximize the surface area, the preparation temperature and time for the MnCo_2_O_4_ were selected as 800 °C and 90 min, respectively. The physical properties of the MnCo_2_O_4_ product were analyzed to determine the metal ion and bond states, elemental distribution, and surface information of the material, which facilitated the subsequent analysis of the microwave absorption mechanism.

**Figure 5:**
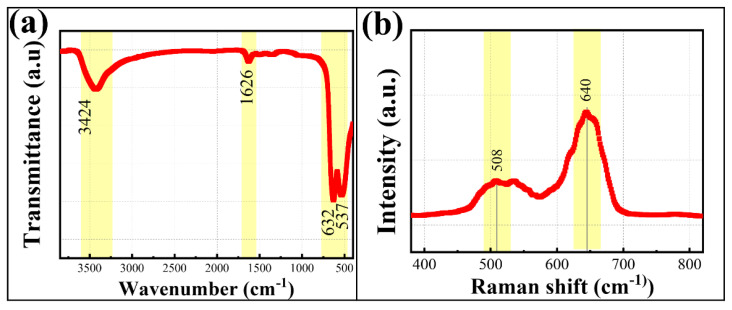
FT-IR (**a**) and Raman (**b**) spectra of calcined products of cobalt-rich slag (800 °C, 90 min).

The metal bond structure and the positions of the metal atoms in the spinel cobalt manganate material prepared by the solid-phase reaction of cobalt-rich slag were determined using FT-IR and Raman methods. As shown in Figure 5a, three distinct infrared absorption peaks appear in the FT-IR spectrum of the sample. The absorption peaks at 537 cm^−1^ and 632 cm^−1^ indicate the formation of Me-O bonds (Me = Mn, Co, Zn, Cd) in the calcined products, which are due to the stretching vibrations of metal atoms in the tetrahedral and octahedral positions of the spinel structure of MnCo_2_O_4_ [30]. In the spectrum, the absorption peaks at 1626 cm^−1^ and 3424 cm^−1^ are attributed to impurities and water molecules attached to the sample surface, respectively. The Raman spectrum is used to analyze the positions of metal atoms in the spinel structure of the material, as shown in Figure 5b. The Raman absorption peaks of MnCo_2_O_4_ appear at 508 cm^−1^ and 640 cm^−1^, indicating the presence of A_1g_ and E_g_ bands related to the combination of tetrahedral and octahedral positions in the material [31]. This confirms that the metal atoms in MnCo_2_O_4_ are distributed at the centers of the tetrahedral and octahedral positions.

**Figure 6:**
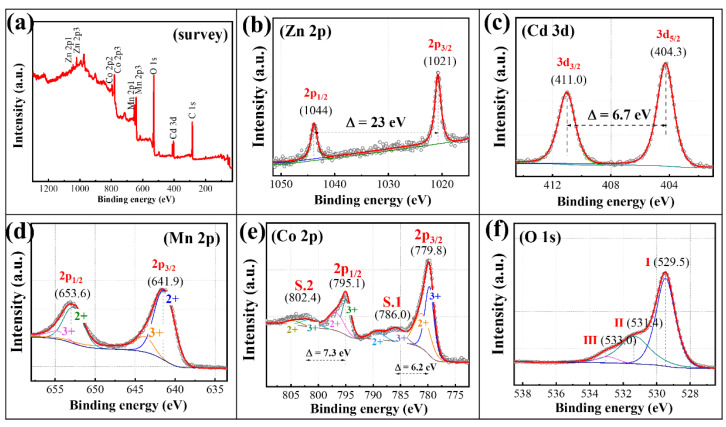
XPS spectra of cobalt-rich slag calcined at 800°C for 90 min: (**a**) Survey spectrum, (**b**) Zn 2p, (**c**) Cd 3d, (**d**) Mn 2p, (**e**) Co 2p, (**f**) O 1s.

The ionic states of the MnCo_2_O_4_ prepared by the solid-phase reaction of cobalt-rich slag are shown in Figure 6. From the full-spectrum XPS of the sample, it can be observed that in addition to the non-metallic elements C and O, the absorption peaks of the metallic elements Mn 2p, Co 2p, Zn 2p, and Cd 3d also exhibit significant absorption intensities in the calcined products. Further peak fitting of the absorption peaks of each metallic element was conducted to determine the oxidation states of the metallic elements in the products. The Zn 2p has distinct spin–orbit splitting peaks at binding energies of 1021 eV and 1044 eV, with a binding energy difference of 23.0 eV, indicating that the Zn element in the calcined products of cobalt-rich slag is entirely in the +2 oxidation state. Similarly, the spin–orbit splitting peaks of Cd 3d, Cd 3d_3/2_, and Cd 3d_5/2_ have a binding energy difference of 6.7 eV, indicating that the Cd element in the products is also entirely in the +2 oxidation state. The fitting results of Mn 2p and Co 2p are similar, indicating that the manganese and cobalt elements in the calcined products coexist in the +2 and +3 oxidation states. Through the analysis of the existence states of the metal ions in the calcined products of cobalt-rich slag, it can be seen that the oxidation forms of the metal elements in the cobalt slag do not change before and after calcination. This suggests that during the solid-phase reaction process, the different components in the cobalt-rich slag mainly undergo metal bond cleavage and recombination to form mixed spinel cobalt manganate.

**Figure 7:**
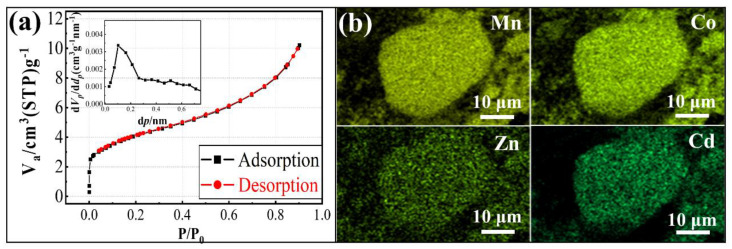
N_2_ adsorption–desorption isotherms (insert is pore size distribution) (**a**) and EDS mapping (**b**) spectra of calcined products of cobalt-rich slag (800 °C, 90 min).

The surface state and elemental distribution of MnCo_2_O_4_ were also tested. From the N_2_ isothermal adsorption–desorption test results, it can be observed that the isothermal adsorption–desorption curve of the calcined product is a Type II adsorption–desorption curve (Figure 7a), indicating that MnCo_2_O_4_ is a non-porous material. The specific surface area and average particle size of the pores were calculated using the BET and BJH models, and are 14.879 m^2^/g and 1.4952 nm, respectively. The EDS mapping results of the calcined products (Figure 7b) show that the metal elements Mn, Co, Zn, and Cd are distributed very uniformly, indicating that during the formation of MnCo_2_O_4_ from cobalt-rich slag, Zn and Cd participate in the solid-phase reaction simultaneously, doping into the spinel lattice in the form of atomic substitution, ultimately forming a homogeneous mixed-type spinel MnCo_2_O_4_.

#### 3.2.3. Physical Property Analysis of MC@G Composites

**Figure 8:**
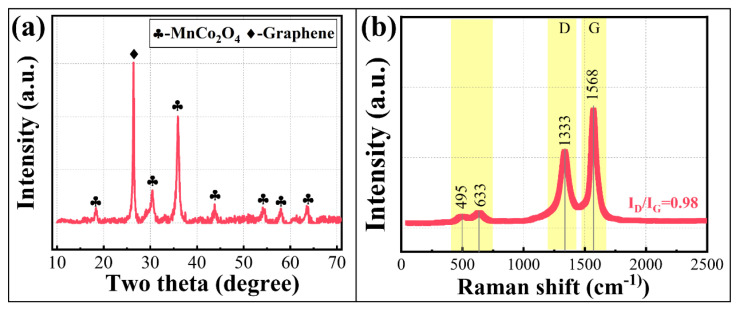
XRD pattern (**a**) and Raman (**b**) spectrum of MC@G composites.

To achieve the materialization application of MnCo_2_O_4_ prepared from cobalt-rich slag, the microwave absorption capability was considered for enhancement [32]. Both ball milling and graphene composite processes were found to reinforce the absorption properties of MnCo_2_O_4_. The ball milling process effectively improves the lattice and morphological state of the material, while the graphene composite process directly enhances the dielectric properties, thereby strengthening the absorption capability. The phase composition and bond structure of the MC@G are shown in Figure 8.

In Figure 8a, the phases of the MC@G are composed of MnCo_2_O_4_ and graphene, with no new phases formed during the compounding process, indicating that the original structures of MnCo_2_O_4_ and graphene are not disrupted. Concurrently, the Raman absorption spectrum of the composites (Figure 8b) exhibits distinct absorption peaks at 495 cm^−1^, 633 cm^−1^, 1333 cm^−1^, and 1568 cm^−1^. From these observations, it can be inferred that the composites maintain Me-O bonds (Me = Mn, Co, Zn, Cd), and the carbon component within the composite material retains a certain degree of disordered structure [33].

**Figure 9:**
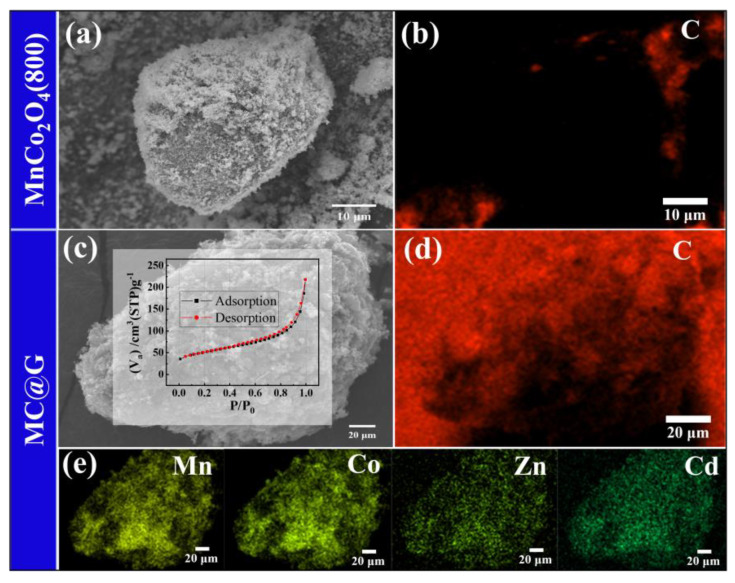
SEM spectra (**a**,**b**), EDS mapping of C element (**c**,**d**) and metal elements (**e**) of MnCo_2_O_4_ (800 °C, 90 min) and MC@G composites (insert is N_2_ adsorption–desorption isotherm of composites).

MnCo_2_O_4_ materials and MC@G composites were subjected to SEM-EDS mapping tests, with the results shown in Figure 9. From the distribution of carbon elements on the material surface before and after compounding, it can be observed that after compounding (Figure 9d), the material surface is covered with a large amount of C elements. This indicates that the graphene compounding process alters the surface state of the material, which is conducive to the effective absorption of microwaves within the material. The distribution of metal elements in MnCo_2_O_4_ after compounding is shown in Figure 9e. Since the compounding process does not change the lattice and atomic structure of the material, the metal elements maintain a uniform distribution. During the ball milling process of MnCo_2_O_4_ and graphene, the objective of encapsulating MnCo_2_O_4_ particles with graphene microparticles was achieved, resulting in the formation of a core–shell structured composite material of MnCo_2_O_4_-graphene. Additionally, after being composited with graphene, the specific surface area of MC@G significantly increased compared to that of MnCo_2_O_4_, rising to a BET surface area of 169.7 m^2^/g.

### 3.3. Microwave Absorption Applications

#### 3.3.1. MnCo_2_O_4_

**Figure 10:**
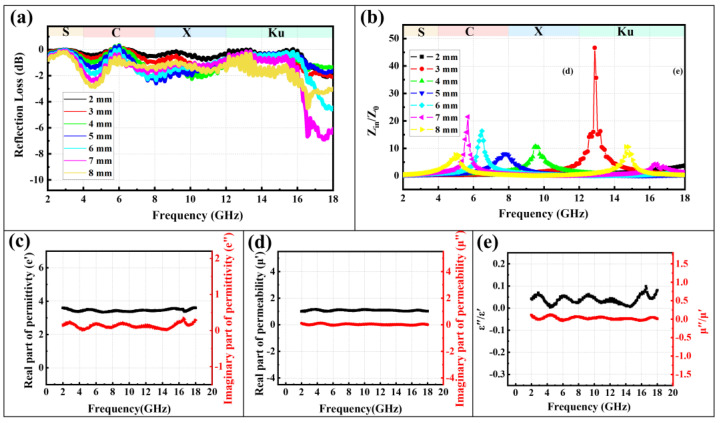
Reflection loss (**a**), Z_in_/Z_0_ (**b**), *ε*′ and *ε*″ (**c**), *μ*′ and *μ*″ (**d**), and *ε*″/*ε*′ and *μ*″/*μ*′ (**e**) results of MnCo_2_O_4_ (800 °C, 90 min).

The microwave absorption performance of the MnCo_2_O_4_ produced from the cobalt-rich slag was tested. The reflection loss values of the materials in the frequency range of 1 to 18 GHz are shown in Figure 10a. Within the matching thickness range of 2 to 8 mm, the minimum reflection loss of the MnCo_2_O_4_ materials does not meet the standard for microwave absorption materials (−10 dB), indicating that MnCo_2_O_4_ prepared by solid-phase reaction cannot be directly used as a microwave absorption material. Further analysis of the limitations of the absorption performance revealed that the impedance matching (Z_in_/Z_0_) performance (Figure 10b) was suboptimal, which directly affected the absorption of microwaves and the dissipation of electromagnetic energy by the MnCo_2_O_4_. Electromagnetic parameters are key indicators for revealing the absorption mechanisms of microwave absorption materials. As shown in Figure 10c–e, MnCo_2_O_4_ prepared from cobalt slag by solid-phase reaction has relatively small real parts (*ε*′) and imaginary parts (*ε*″) of the dielectric constant, with only minor fluctuations in the dielectric tangent value. The real parts (*μ*′) and imaginary parts (*μ*″) of the permeability, as well as the permeability tangent value, remain essentially unchanged. Therefore, it can be determined that MnCo_2_O_4_ prepared from cobalt slag by solid-phase reactions possesses a certain dielectric loss capability for microwaves, but has poor magnetic loss capability [34].

#### 3.3.2. MC@G Composites

In order to optimize the impedance matching and reflection loss attributes of the MnCo_2_O_4_, a ball milling composite process utilizing graphene materials was employed. Leveraging the characteristic two-dimensional structure of graphene, multiple reflection pathways for electromagnetic waves on the surface of MnCo_2_O_4_ were constructed [35].

**Figure 11:**
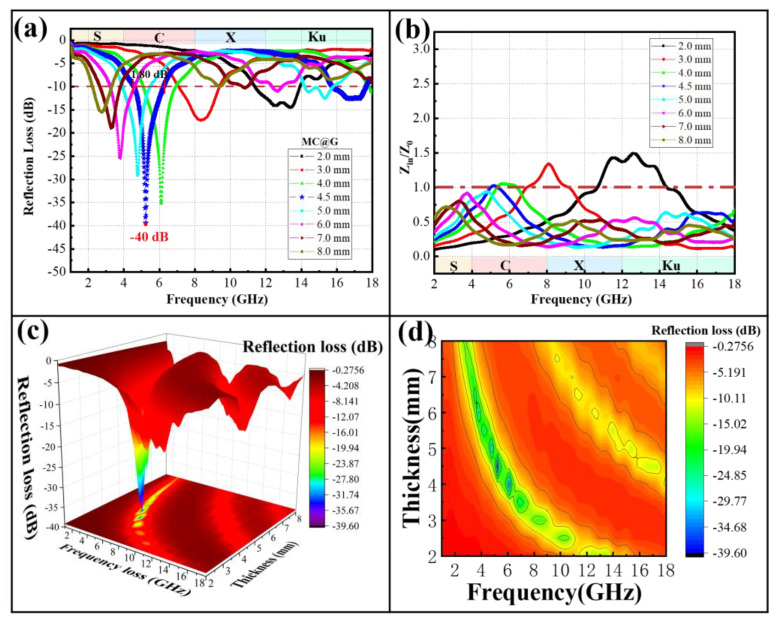
Reflection loss (**a**), Z_in_/Z_0_ (**b**), and 3D and 2D reflection loss (**c**,**d**) of MC@G composites.

The reflection loss results of the MC@G composites within the 1 to 18 GHz frequency range are depicted in Figure 11a,c. The minimum reflection loss of the composites exhibits a trend of initially increasing and then decreasing. When the coating thickness is 4.5 mm, the reflection loss reaches its lowest point of −40 dB at 5.2 GHz, with an effective absorption bandwidth of 1.8 GHz. Furthermore, Figure 11d reflects that as the thickness increases, the effective absorption band of the composites shifts towards lower frequencies and the corresponding effective absorption bandwidth gradually decreases. Impedance matching is a crucial factor in evaluating the absorption performance of materials. As shown in Figure 11b, the Z_in_/Z_0_ of the MnCo_2_O_4_ materials is significantly improved after compounding. At a matching thickness of 4.5 mm, Z_in_/Z_0_ reaches 1, indicating that the MC@G achieves the optimal balance between the absorption and energy dissipation of microwaves at this point, allowing the material to absorb microwave energy to the greatest extent and convert it into other forms of energy, such as thermal energy, within its structure.

**Figure 12:**
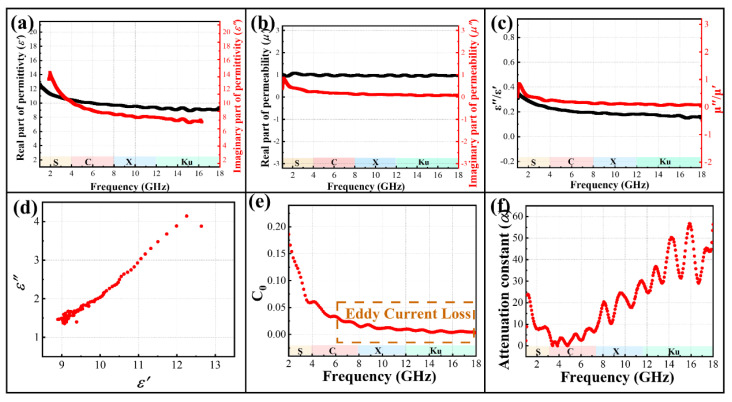
*ε*′ and *ε*″ (**a**), *μ*′ and *μ*″ (**b**), *ε*″/*ε*′ and *μ*″/*μ*′ (**c**), Cole-Cole curves (**d**), C_0_ (**e**) and attenuation constant (**f**) of MC@G composite.

The electromagnetic parameters of the MC@G are shown in Figure 12a–c. Compared to the electromagnetic parameters of MnCo_2_O_4_ (Figure 10c–e), the *ε*′ and *ε*″ of the composite material are significantly enhanced, and the decreasing trend with increasing frequency is also very evident. This indicates that the composite material has a stronger capacity for storing and dissipating microwaves, demonstrating good dielectric loss capabilities for microwaves. In Figure 12a, oscillation peaks appear in the X and Ku bands, which are attributed to the interfacial polarization effect caused by the accumulation of charges at the heterojunctions between different components in the MC@G. Moreover, after ball milling and compounding, the MnCo_2_O_4_ materials contain a large number of defects and dipoles, which are conducive to the dissipation of microwaves. Figure 12d shows the Cole–Cole plot of the MC@G, and the appearance of the Cole semicircle further indicates that the dielectric loss of the MC@G is primarily due to relaxation polarization occurring in the MC@G under alternating electromagnetic fields [36]. Additionally, the latter part of the sample curve gradually becomes a straight line, indicating that the material also possesses a certain degree of conductive loss capability. On the other hand, the *μ′* and *μ″* of the composites do not show significant improvement, and the tangent value of the permeability maintains the variation trend of the *μ″* of the permeability, indicating the poor magnetic loss capability of the composites. Eddy current loss is a common phenomenon of magnetic hysteresis loss (Figure 12e), and it can be observed that as the frequency increases, the decreasing trend of the C_0_ value gradually diminishes, indicating that the composite material also has a certain eddy current loss capability during the microwave absorption process. The attenuation constant represents the magnitude of the material’s energy dissipation capability for microwaves, with a larger value indicating a greater energy dissipation capability. Figure 12e shows that as the frequency increases, the fluctuation of the attenuation constant of the composites also increases, with the maximum attenuation constant value reaching 58.

In summary, it can be determined that the MC@G primarily absorbs microwaves through its dielectric loss capabilities. The MC@G powder prepared in this study exemplifies this mechanism and is particularly suitable for the preparation of coating-type absorbing materials. For instance, in China’s wind power industry, where anti-corrosion absorbing coatings are required, the industry standard (T/CANSI 150-2024) mandates that the reflection loss of such coatings must be ≤−10 dB. The MC@G developed here achieves a reflection loss of −40 dB at 5.2 GHz (with a coating thickness of 4.5 mm) and an effective absorption bandwidth of 1.8 GHz, thereby meeting the standard and qualifying as an effective component for commercial absorbing coating formulations. Table 2 lists some absorbing materials reported in the literature, and it can be seen that the absorbing properties of the MC@G prepared by the solid-phase method are comparable to those of the materials prepared by the hydrothermal method.

## 4. Conclusions

In summary, we successfully prepared MnCo_2_O_4_ materials from cobalt-rich slag through a solid-state reaction method. The results showed that the formation of MnCo_2_O_4_ was achieved at a calcination temperature of 800 °C and a duration of 90 min. Building on this, we further prepared MC@G composites using ball milling and composite techniques, which significantly enhanced the microwave absorption properties of the material. The minimum reflection loss of the MC@G composites reached −40 dB at 5.2 GHz, with an effective absorption bandwidth of 1.8 GHz when the coating thickness was 4.5 mm. The MC@G composites exhibit excellent microwave absorption properties, making them promising candidates for microwave-absorbing applications. This study provides a novel approach for the value-added utilization of valuable metal resources in cobalt-rich slag.

## Figures and Tables

**Table 1:** The metal element contents (wt.%) of the cobalt-rich slag before and after washing, as determined by ICP-AES.

Sample	Element Contents (wt.%)				
	Co	Mn	Zn	Cd	Cu
Cobalt-Rich Slag Before Washing	22.01	19.64	6.19	3.41	0.03
Cobalt-Rich Slag After Washing	21.08	19.04	3.11	2.68	0.03

**Table 2:** Comparison of MC@G with reported absorbing materials.

Materials	RL_min_ [dB]/Thickness [mm]	EBA [GHz]/Thickness [mm]	References
MC@G	−40 dB/4.5	5.2 GHz/4.5	This Study
Fe_3_O_4_@polypyrrole	−41.9 dB/2.0	6.0 GHz/2.0	[37]
Fe-HPCNFs	−46.9 dB/2.0	3 GHz/2.0	[38]
MCF/Co	−40.1 dB/3.0	6.24 GHz/3.0	[39]
CoZnO/C@BCN	−54.9 dB/3.8	5.2 GHz/1.9	[40]
Polyimide-based graphene foam	−61.29 dB/4.75	5.51 GHz/4.75	[41]

## Data Availability

The original contributions presented in this study are included in the article. Further inquiries can be directed to the corresponding author.

## Nomenclature

The following abbreviations and units are used in this manuscript:

MC@G	Graphene-encapsulated MnCo_2_O_4_ composites
XRD	X-ray diffraction
SEM	Scanning electron microscope
VSM	Vibrating sample magnetometer
FT-IR	Fourier transform infrared spectrometer
XPS	X-ray photoelectron spectroscopy
BET	Brunauer–Emmett–Teller theory
BJH	Barret–Joyner–Halenda theory
Z_in_/Z_0_	Impedance matching performance
ε′	Real parts of the dielectric constant
ε″	Imaginary parts of the dielectric constant
μ′	Real parts of the permeability
μ″	Imaginary parts of the permeability
dB	Decibel
°C	Celsius
min	Minute
cm	Centimeter
mm	Millimeter
nm	Nanometer
kV	Kilovolt
eV	Electronvolt
GHz	Gigahertz
